# MGOGP: a gene module-based heuristic algorithm for cancer-related gene prioritization

**DOI:** 10.1186/s12859-018-2216-0

**Published:** 2018-06-05

**Authors:** Lingtao Su, Guixia Liu, Tian Bai, Xiangyu Meng, Qingshan Ma

**Affiliations:** 1 College of Computer Science and Technology, Jilin University, Changchun, 130012 China; 2 Key Laboratory of Symbolic Computation and Knowledge Engineering of Ministry of Education, Jilin University, Changchun, 130012 China; 3 The First Clinical Hospital of Jilin University, Changchun, 130021 China

**Keywords:** Gene prioritization, Gene module, Gene ontology, Cancer-related genes

## Abstract

**Background:**

Prioritizing genes according to their associations with a cancer allows researchers to explore genes in more informed ways. By far, Gene-centric or network-centric gene prioritization methods are predominated. Genes and their protein products carry out cellular processes in the context of functional modules. Dysfunctional gene modules have been previously reported to have associations with cancer. However, gene module information has seldom been considered in cancer-related gene prioritization.

**Results:**

In this study, we propose a novel method, MGOGP (Module and Gene Ontology-based Gene Prioritization), for cancer-related gene prioritization. Different from other methods, MGOGP ranks genes considering information of both individual genes and their affiliated modules, and utilize Gene Ontology (GO) based fuzzy measure value as well as known cancer-related genes as heuristics. The performance of the proposed method is comprehensively validated by using both breast cancer and prostate cancer datasets, and by comparison with other methods. Results show that MGOGP outperforms other methods, and successfully prioritizes more genes with literature confirmed evidence.

**Conclusions:**

This work will aid researchers in the understanding of the genetic architecture of complex diseases, and improve the accuracy of diagnosis and the effectiveness of therapy.

**Electronic supplementary material:**

The online version of this article (10.1186/s12859-018-2216-0) contains supplementary material, which is available to authorized users.

## Background

Discovering cancer-related genes has profound applications in modelling, diagnosis, therapeutic intervention, and in helping researchers get clues on which genes to explore [1–3]. Computational approaches are preferred due to their high efficiency and low cost [4, 5]. Many computational methods have been proposed, including: a) gene-based function similarity measure methods [6–9]; b) biological interaction network-based methods [10–14], and c) methods based on multiple datasets fusion [15–17]. Methods of the first kind based on the hypothesis that phenotypically similar diseases are caused by functionally related genes. Based on this hypothesis, many methods prioritize genes by computing similarity scores between the candidate genes and the known disease genes. For example, ToppGene [6] ranks genes based on similarity scores of each annotation of each candidate genes by comparing enriched terms in a given set of training genes. Endeavour [8] prioritizes candidate genes by similarity values between candidate genes and seed genes, by integrating more than six types of genomic datasets from over a dozen data sources. Methods of the second kind prioritize genes using the guilt-by-association principle, which means genes interacting with known disease genes are more likely disease-related genes. For instance, PINTA [10] prioritizes candidate genes by utilizing an underlying global protein interaction network. Other methods rank candidate genes by exploiting either local or global network information [2]. Methods of the last kind incorporate datasets such as gene expression, biomedical literature, gene ontology, and PPIs together for gene prioritization. For example, ProphNet [17] integrates information of different types of biological entities in a number of heterogeneous data networks. Taking all these methods into consideration, they are either gene-centric or network-centric. However, gene module as a basic functional unit of genes has seldom been considered.

Gene module can be defined as a protein complex, a pathway, a sub-network of protein interactions. Module detection has long been studied and many useful algorithms have been proposed, such as [18–21]. Although different methods have different module detection strategies, most of them rely on PPIs network. PPIs network suffers from drawbacks as highlighted in [22]. Firstly, the PPI network is incomplete, which only covers the interactions of well-researched proteins. For instance, of the 20,502 genes in the gene expression matrix downloaded from The Cancer Genome Atlas (TCGA), only 9078 (44.2%) and 2761 (13.4%) genes are included in Human Protein Reference Database (HPRD) [23] and Database of Interacting Proteins (DIP) [24] PPIs networks respectively. As a result, detected modules are incomplete and their accuracy are limited. Secondly, protein interactions in PPIs network suffer from high false positive and negative rates, modules discovered from such PPI data also suffer from high false rates. All these inherent limitations affect the coverage and accuracy of the inferred modules.

Nowadays, numerous public databases of protein and gene annotation information are available, such as Entrez Gene [25], Ensembl [26], PIR iProClass [27], GeneCards [28], KEGG [29], Gene Ontology Consortium [30], DAVID [31], GSEA [32] and UniProt [33]. For instance, DAVID [31] contains information on over 1.5 million genes from more than 65,000 species, with annotation types, including sequence features, protein domain information, pathway maps, enzyme substrates and reaction, protein-protein interaction data and disease associations. Gene Ontology Consortium describes the functions of specific genes, using terms known as GO (Gene Ontology). KEGG map genes to pathways while GSEA provides functional gene groups collected from BioCarta genes sets, KEGG gene sets and Reactome gene sets. With these annotation information, we can easily group genes into functional modules.

Complex diseases, especially cancer are caused by the dysfunction of groups of genes and/or gene interactions rather than the mutations of individual genes. Detecting and prioritizing cancer-related genes from the perspective of gene module is promising. Although some useful work has been conducted [34, 35], the results are still far from being satisfactory. In this study, we take the importance of not only genes but also their affiliated modules into consideration, and prioritizing genes in a heuristic way. We measure module importance by the number of differential genes within the module and the number of differential correlations between the module genes. Besides, the number of known cancer-related genes in the module is also considered. We measure the gene importance by three aspects information: a), gene’s differential expression value, b), the number of differential correlations between the gene and all other module gene. c), the fuzzy measure based similarity values between the gene and all known cancer-related genes (if exist) within the module. The global rank of all genes is obtained by utilizing a rank fusion strategy.

## Methods

**Fig. 1:**
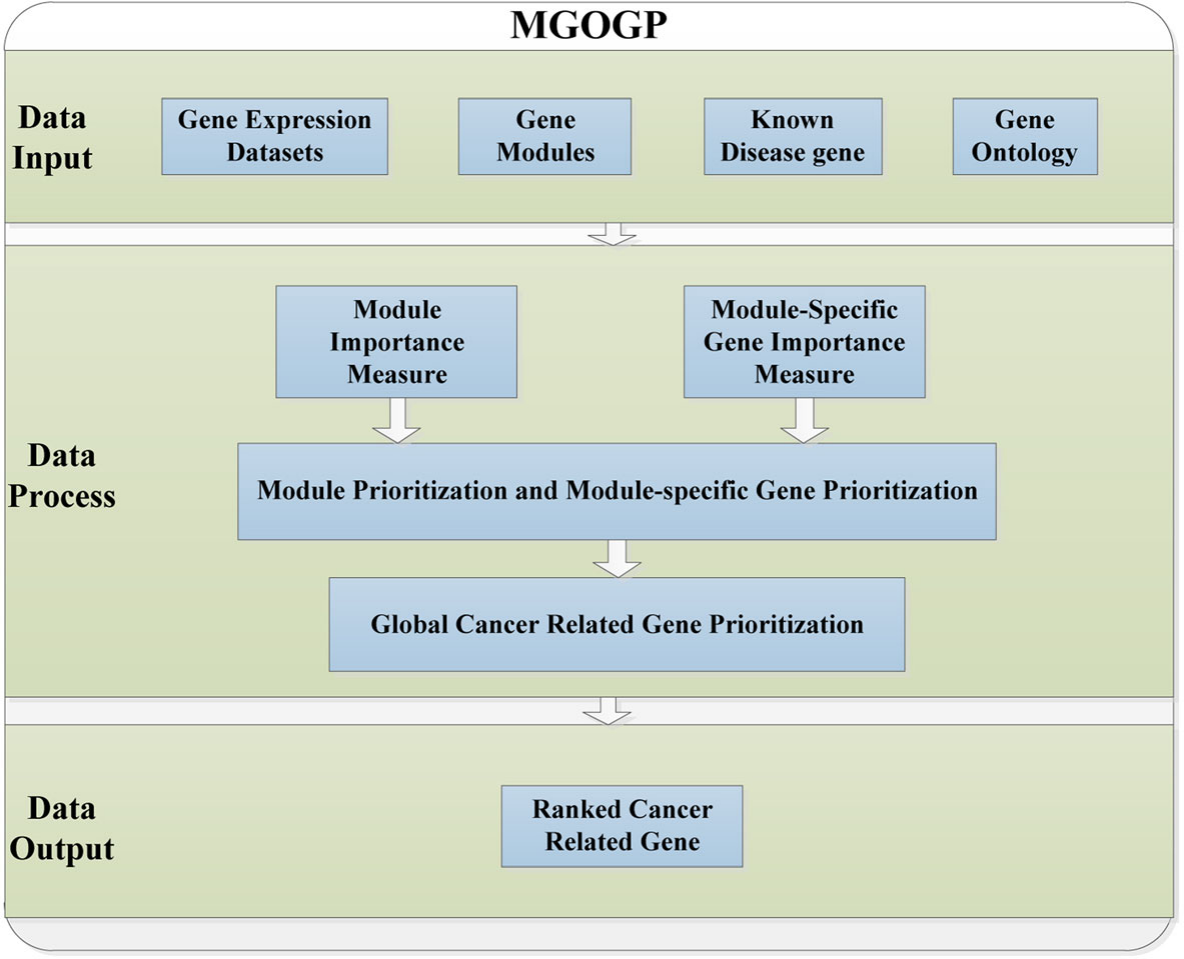
Main components of MGOGP

**Fig. 2:**
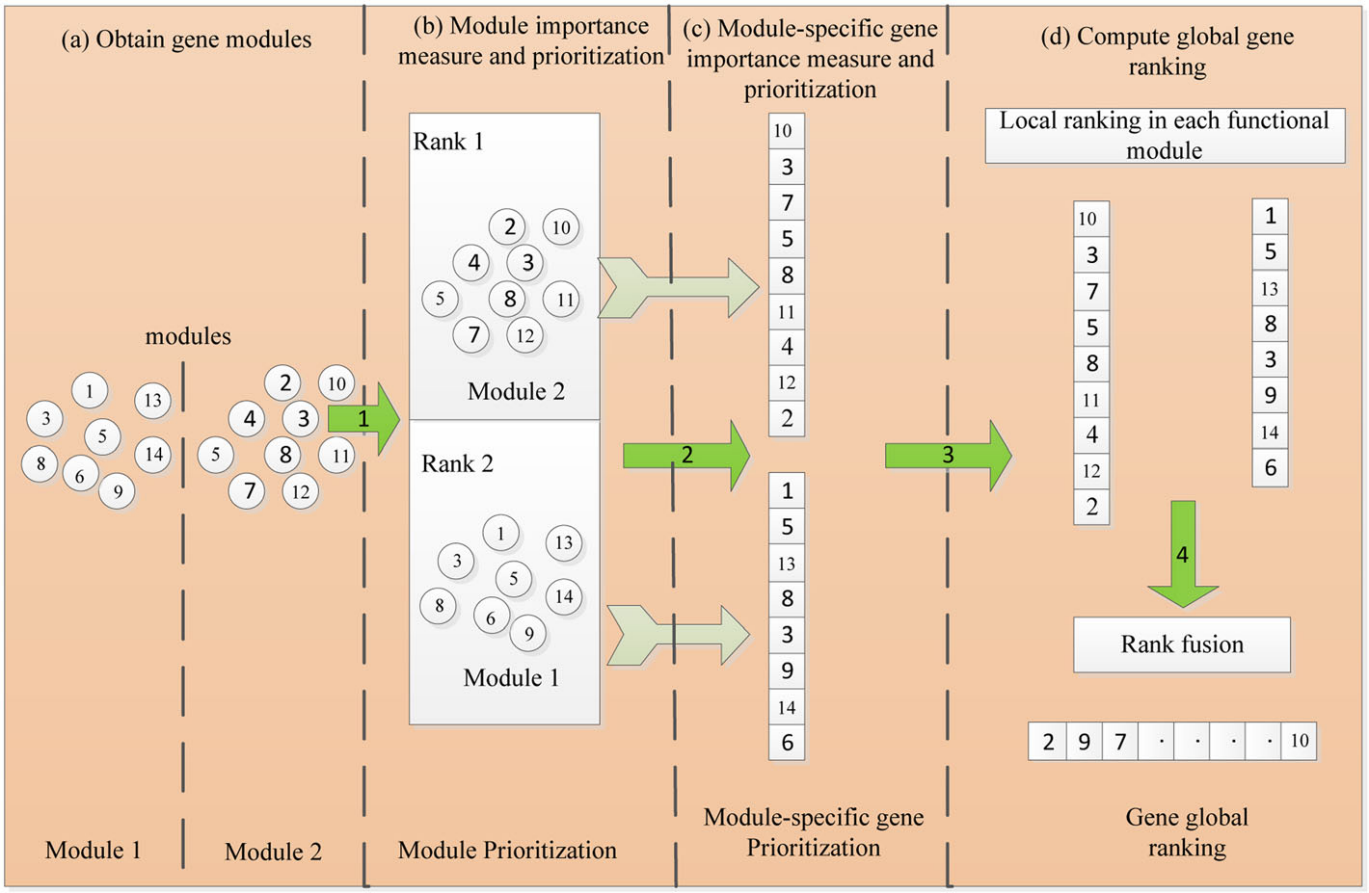
MGOGP processes are illustrated. **a** Obtain gene modules, **b** Module importance measure and prioritization, **c** Module-specific gene importance measure and prioritization, **d** Compute global gene ranking

As shown in Fig.1, MGOGP takes gene expression datasets, gene modules, known disease genes and gene ontology annotation information [36] as input, and the ranked genes as output. The main parts including: module importance measure, module-specific gene importance measure, module rank and module-specific gene prioritization, and global cancer-related gene prioritization. Figure 2 schematically illustrates these steps in detail.

First, obtain functional gene modules; then get the global ranking of all modules and the local ranking of all module-specific genes based on their importance; finally, the rank fusion algorithm further gives all genes a global rank.

### Input datasets

As shown in Fig. 1, MGOGP takes gene expression datasets, gene modules, known disease-related genes and gene ontology annotation information as input. In this study, all gene modules are downloaded from GSEA website (http://software.broadinstitute.org/gsea/downloads.jsp). All GO ontologies of genes are downloaded from GeneCards [37, 38]. Information of relationships between GO terms are got from Gene Ontology Consortium website.

### Module importance measure

We measure the importance of a module by: the number of differentially expressed genes in the module, the number of differential correlations between module genes and the basic importance of the module itself.

We use DESeq2 for gene differential expression analysis [3, 35, 39, 40]. If genes with *padj*(*g*_*i*_) value bigger than the threshold value *μ*, we set *Se*(*g*_*i*_) = 0. Otherwise, we set *Se*(*g*_*i*_) = 1, which means the gene *g*_*i*_ is a candidate differential expression gene. *Se*(*g*_*i*_) is defined as follows:

1 $$ Se\left({g}_i\right)=\left\{\begin{array}{l}0,\mathrm{if}\; padj\left({g}_i\right)>\mu \\ {}1,\mathrm{else}\end{array}\right. $$

To further improve the statistical significance of the selected candidate differential expression genes, we applied a multiple random sampling strategy. As defined in Eq. 2.

2 $$ DEG\left({g}_i\right)=\left\{\begin{array}{l}0,\mathrm{if}\frac{1}{s}\sum \limits_{s=1}^S Se\left({g}_i\right)<\omega \\ {}1,\mathrm{else}\end{array}\right. $$

Where *S* is the number of sampling; *ω* is a threshold value; if a gene *g*_*i*_ is selected as a differential expression gene we set *DEG*(*g*_*i*_) = 1, Otherwise, we set *DEG*(*g*_*i*_) = 0.

We define *Ncr*(*m*_*j*_) as the ratio of differential expression genes in the module *m*_*j*_ as shown in Eq. 3:

3 $$ {\displaystyle \begin{array}{l} Ncr\left({m}_j\right)=\frac{\sum_{i=1}^N DEG\left({g}_i\right)}{N}\\ {}j\in 1,2,3,\dots, M\end{array}} $$

Where, *g*_*i*_ is the *i*th gene in the module *m*_*j*_; *N* is the total number of genes in the module *m*_*j*_; *DEG*(*g*_*i*_) is defined in Eq. 2.

Next, for each pair of genes in the module *m*_*j*_, two correlation values are calculated using normal and tumor samples respectively. As defined in Eqs. 4 and 5 respectively.

4 $$ {r}_N\left({g}_i,{g}_h\right)=\frac{\sum_{l=1}^L\left({x}_l-\overline{x}\right)\left({y}_l-\overline{y}\right)}{\sqrt{\sum_{l=1}^L{\left({x}_l-\overline{x}\right)}^2{\left({y}_l-\overline{y}\right)}^2}} $$

*r*_*N*_(*g*_*i*_, *g*_*h*_) is the Pearson correlation value between gene *g*_*i*_ and gene *g*_*h*_ across all normal samples. *L* is the normal sample number.

5 $$ {r}_T\left({g}_i,{g}_h\right)=\frac{\sum_{q=1}^Q\left({x}_q-\overline{x}\right)\left({y}_q-\overline{y}\right)}{\sqrt{\sum_{q=1}^Q{\left({x}_q-\overline{x}\right)}^2{\left({y}_q-\overline{x}\right)}^2}} $$

*r*_*T*_(*g*_*i*_, *g*_*h*_) is the Pearson correlation value between gene *g*_*i*_ and gene *g*_*h*_ across all tumor samples. *Q* is the tumor sample number.

To test whether the correlation coefficient between gene *g*_*i*_ and gene *g*_*h*_ is differentially correlated, we test whether *r*_*T*_(*g*_*i*_, *g*_*h*_) and *r*_*N*_(*g*_*i*_, *g*_*h*_) are significantly different. The two correlation coefficients are changed to *Z*_*N*_(*g*_*i*_, *g*_*h*_) and *Z*_*T*_(*g*_*i*_, *g*_*h*_) respectively.

6 $$ {Z}_N\left({g}_i,{g}_h\right)=\frac{1}{2}\log \frac{1+{r}_N\left({g}_i,{g}_h\right)}{1-{r}_N\left({g}_i,{g}_h\right)} $$

Similarly, *r*_*T*_(*g*_*i*_, *g*_*h*_) is changed to *Z*_*T*_(*g*_*i*_, *g*_*h*_) as Eq. (6). The differential correlation is tested based on Fisher’s z-test [41]. As defined in Eq. (7):

7 $$ Z=\frac{Z_N\left({g}_i,{g}_h\right)-{Z}_T\left({g}_i,{g}_h\right)}{\sqrt{\frac{1}{L-3}+\frac{1}{Q-3}}} $$

The *Z* value has an approximately Gaussian distribution under the null hypothesis [41]. If the *fdr* value of a gene is bigger than the threshold value *υ*, we set *Sc*(*g*_*i*_, *g*_*h*_) = 0, otherwise we set *Sc*(*g*_*i*_, *g*_*h*_) = 1, which means the correlation coefficient is a potential differential correlation. *Sc*(*g*_*i*_, *g*_*h*_) is defined as follows:

8 $$ Sc\left({g}_i,{g}_h\right)=\left\{\begin{array}{l}0,\mathrm{if}\; fdr\left({g}_i,{g}_h\right)>\upsilon \\ {}1,\mathrm{else}\end{array}\right. $$

Where *fdr*(*g*_*i*_, *g*_*h*_) is the local false-discovery rate (*fdr*) derived from fdrtool package [42]; *υ* is a threshold value.

As the way we find differential expression genes, we retain only those significantly changed correlations. As defined in Eq. 9:

9 $$ DEE\left({g}_i,{g}_h\right)=\left\{\begin{array}{l}0,\mathrm{if}\frac{1}{s}\sum \limits_{s=1}^S Sc\left({g}_i,{g}_h\right)<\delta \\ {}1,\mathrm{else}\end{array}\right. $$

Where *S* is the number of sampling; *δ* is a threshold value; we set *DEE*(*g*_*i*_, *g*_*h*_) = 1 if the gene *g*_*i*_ and *g*_*h*_ are differentially correlated. Otherwise, we set *DEE*(*g*_*i*_, *g*_*h*_) = 0.

We define *Ecr*(*m*_*j*_) as the ratio of differential correlations among genes in the module *m*_*j*_. *Ecr*(*m*_*j*_) is defined in Eq. 10:

10 $$ {\displaystyle \begin{array}{l} Ecr\left({m}_j\right)=\frac{\sum_{k=1}^K DEE\left({g}_i,{g}_h\right)}{K}\\ {}K=\frac{N\left(N-1\right)}{2}\; and\;i,h\in 1,2,3,\dots, N\end{array}} $$

*K* and *N* is the edge number and the gene number of the module *m*_*j*_ respectively.

We measure the basic importance of a module by calculating the ratio of known disease genes in a module, as shown in Eq. 11:

11 $$ info\left({m}_j\right)=\left( num\left({d}_j\right)+1\right)/N $$

*num*(*d*_*j*_) is the number of known disease genes in the module *m*_*j*_; *N* is the number of genes in the module *m*_*j*_.

The module importance is defined in Eq. 12.

12 $$ {\displaystyle \begin{array}{l}p\left({m}_j\right)=\left(\left( Ncr\left({m}_j\right)+ Ecr\left({m}_j\right)\right)/2\right)\ast info\left({m}_j\right)\ \\ {}\ \mathrm{j}\in 1,2,3\dots, \mathrm{M}\end{array}} $$

where *m*_*j*_ means the *j*th module; *M* is the total number of modules.

### Module-specific gene importance measure

We measure the importance of a gene (*p*(*g*_*i*_)) in the module by measuring: the gene’s differential expression value, the number of differential correlations between the gene and all other module genes and the basic importance of the gene itself.

The number of differential correlations (*CorC*(*g*_*i*_)) between the gene *g*_*i*_ and all other genes in the same module is calculated as in Eq. 13.

13 $$ {\displaystyle \begin{array}{l} CorC\left({g}_i\right)=\frac{\sum_{h=1,h\ne i}^{N-1} Sc\left({g}_i,{g}_h\right)}{N-1}\\ {}i,h\in 1,2,3,\dots, N,{g}_i\in {m}_j\\ {}j\in 1,2,3,\dots, M\end{array}} $$

*N* is the number of genes in the module *m*_*j*_; *M* is the total module number.

Finally, the basic importance of a gene is determined by the gene ontology-based fuzzy measure similarity values between the gene and all known disease gene (if exist) in the same module. As shown in Eq. 14.

14 $$ {\displaystyle \begin{array}{l} info\left({m}_j\_{g}_i\right)=\\ {}\left\{\begin{array}{l}0, if\; num\left({m}_j\_{d}_h\right)=0\\ {}1, if\;{g}_i\; is\;a\; known\kern0.17em disease\kern0.17em gene\kern0.17em itself\\ {}{\sum}_{h=1}^{num\left({m}_j\_{d}_h\right)}{S}_{FMS}\left({g}_i,{m}_j\_{d}_h\right)/ num\left({m}_j\_{d}_h\right), else\end{array}\right.\end{array}} $$

*num*(*m*_*j*__*d*_*h*_) is the number of known disease genes in the module *m*_*j*_. If *num*(*m*_*j*__*d*_*h*_) = 0, which means no known disease gene in the module *m*_*j*_, we set *info*(*m*_*j*__*g*_*i*_) = 0. If *g*_*i*_ itself is a known disease gene, we set *info*(*m*_*j*__*g*_*i*_) = 1. Otherwise, we calculate the gene importance value based on the fuzzy similarity measure between the gene and all the known disease gene in the module *m*_*j*_. *S*_*FMS*_(m_*j*__*g*_*i*_, m_*j*__*d*_*h*_) is defined in Eq. 15, as in [43]:

15 $$ {S}_{FMS}\left({\mathrm{m}}_j\_{g}_i,{\mathrm{m}}_j\_{d}_h\right)=\frac{Sm_i\left({T}_{{\mathrm{m}}_j\_{g}_i}\cap {T}_{m_j\_{d}_h}\right)+{Sm}_h\left({T}_{{\mathrm{m}}_j\_{g}_i}\cap {T}_{m_j\_{d}_h}\right)}{2} $$

Where *Sm*_*i*_ is the Sugeno measure [43] defined on GO terms of gene m_*j*__*g*_*i*_ and *Sm*_*h*_ is the Sugeno measure defined on GO terms of module disease gene *m*_*j*__*d*_*h*_.

Let ${T}_{{\mathrm{m}}_j\_{g}_i}$ is the set of GO annotation terms of gene m_*j*__*g*_*i*_, *Sm*_*i*_, is a real value function, satisfying [44]:

1. $$ {Sm}_i\left({T}_{{\mathrm{m}}_j\_{g}_i}\right)=0, if\;{T}_{{\mathrm{m}}_j\_{g}_i}=\varnothing, else\;{Sm}_i\left({T}_{{\mathrm{m}}_j\_{g}_i}\right)=1. $$
2. $$ {Sm}_i\left({T}_{{\mathrm{m}}_j\_{g}_i}\right)\le Sm\left({T}_{m_j\_{d}_h}\right)\; if\;{T}_{{\mathrm{m}}_j\_{g}_i}\subseteq {T}_{m_j\_{d}_h} $$
3. For all ${T}_A,{T}_B\subseteq {T}_{{\mathrm{m}}_j\_{g}_i}$ with *T*_*A*_ ∩ *T*_*B*_ = Φ
$$ {\displaystyle \begin{array}{l}{Sm}_i\left({T}_A\cup {T}_B\right)={Sm}_i\left({T}_A\right)+{Sm}_i\left({T}_B\right)\\ {}+\lambda {Sm}_i\left({T}_A\right){Sm}_i\left({T}_B\right),\lambda >-1\end{array}} $$

For a given gene annotation set ${T}_{{\mathrm{m}}_j\_{g}_i}$, the parameter *λ* of its Sugeno fuzzy measure can be uniquely solved as in Eq. 16:

16 $$ \left(1+\lambda \right)=\prod \limits_{i=1}^n\left(1+\lambda {Sm}_i\right) $$

This equation has a unique solution for *λ* > −1. Let *Sm*_*k*_ = *Sm*({*T*_*k*_}). The mapping *T*_*k*_ → *Sm*_*k*_ is called a fuzzy density function. The fuzzy density value, *Sm*_*k*_, is interpreted as the importance of the single information source *T*_*k*_ in determining the similarity of two genes. As defined in Eq. 17:

17 $$ {Sm}_k=-\ln \left(p\left({T}_k\right)/\underset{T_j\in {T}_{g_i}}{\max}\left\{-\ln \left(p\left({T}_j\right)\right)\right\}\right) $$

Where *p*(*T*_*k*_) is defined in Eq. 18:

18 $$ {\displaystyle \begin{array}{l}p\left({T}_k\right)=\\ {}\left(\frac{count\left({T}_k+ children\kern0.17em of\;{T}_k\; in\kern0.17em corpus\right)}{count\left( all\; GO\; term\mathrm{s}\;\mathrm{in}\; corpus\right)}\right)\\ {}1\le k\le \mid {T}_{g_i}\mid \end{array}} $$

The importance of gene (*p*(*g*_*i*_)) in a module is defined in Eq. 19.

19 $$ {\displaystyle \begin{array}{l}p\left({g}_i\right)= padj\left({g}_i\right)+ CorC\left({g}_i\right)+ info\left({g}_i\right)\\ {}i\in 1,2,3,\dots, N,{g}_i\in {m}_j\end{array}} $$

*N* is the number of genes in the module *m*_*j*_.

### Global gene ranking

Most genes deploy their functions in the context of sophisticated functional modules [45, 46]. Therefore, the global rank of a gene need be decided by its own importance and the importance of its affiliated module. As in [34], a rank fusion strategy is used to fuse the local rank of genes in each module into a global rank.

The rank fusion strategy is a recursive process. It decides the rank of the *nth* gene based on all the top-ranked *n* − 1 genes. We define *i* as the number of genes having already obtained their global ranking in the recursive process of rank fusion, *m*(*i*, *j*) as the number of top *i* genes located in the module *j* after having determined the top *i* genes. *t*(*i*, *j*) as the expectation of the number of top *i* genes located in the module *j*. *e*(*i*, *j*) as the expectation of probability that the *i* + 1 globally ranked genes come from the module *j*. We use the module importance value *p*(*m*_*j*_) as the probability of a disease-related gene comes from it. The relationship between *i*, *m*(*i*, *j*), *t*(*i*, *j*) and *p*(*m*_*j*_) is shown in Eq. 20:

20 $$ {\displaystyle \begin{array}{l}t\left(i,j\right)= ip\left({m}_j\right)\\ {}e\left(i,j\right)=t\left(i+1,j\right)-m\left(i,j\right)\end{array}} $$

**Fig. 3:**
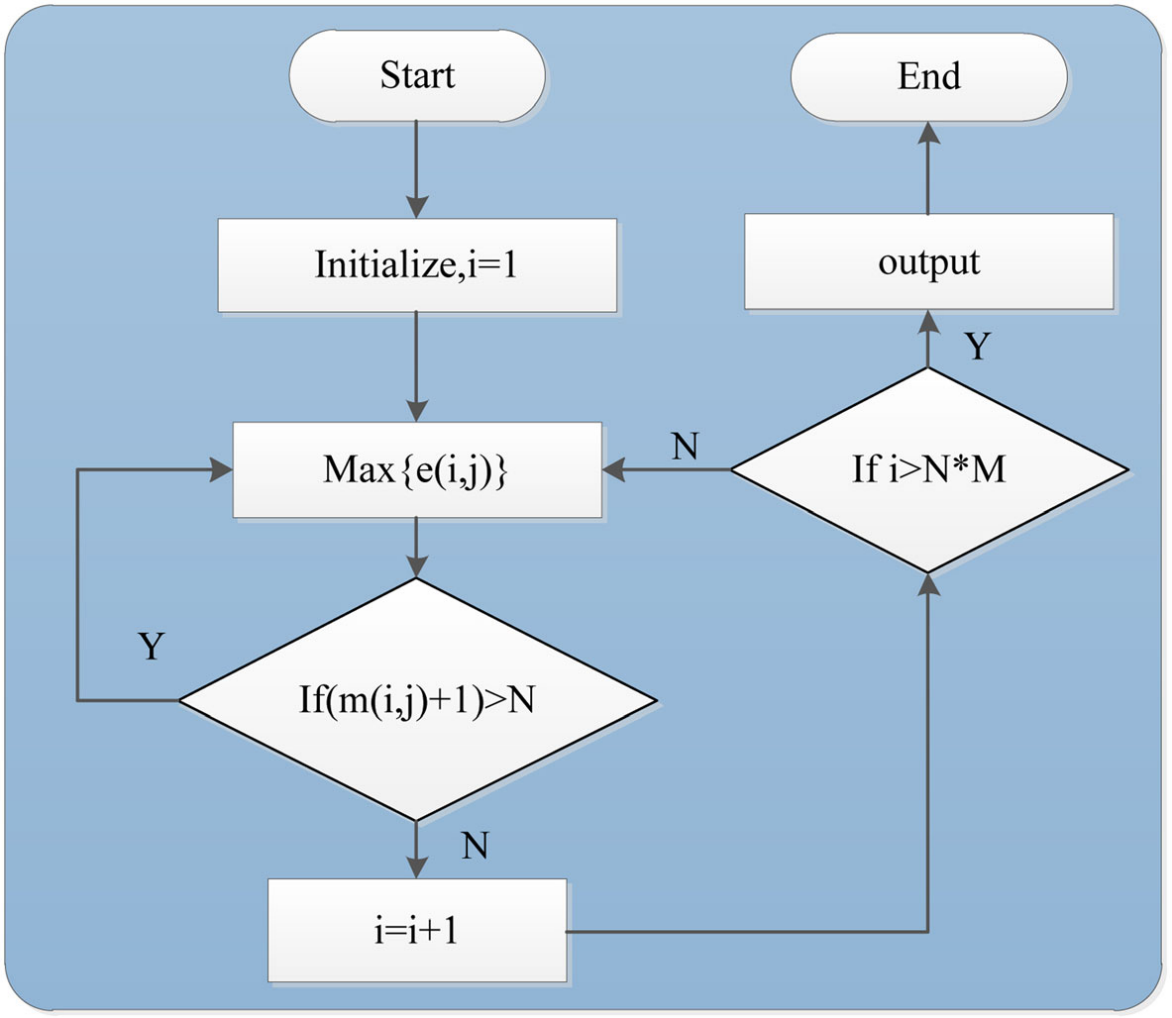
Rank fusion process. *N* is the number of genes in the module *j*, *M* is the total module number

Initially, the first ranked gene in the module with highest importance value is chosen as the top 1 gene in the gene’s global rank, because all genes in each module have been ranked from big to small according to their importance value. Let *i* as the number of genes having obtained their global ranking, to decide the *i* + 1 ranked gene, we need to find the module with the biggest *e*(*i*, *j*) value, because *e*(*i*, *j*) indicates the expectation of probability that the *i* + 1 globally ranked genes from module *j*. So the genes ranked *m*(*i*, *j*) + 1 in the module *j* will be chosen as the top *i* + 1 ranked gene, because in the module *j*, top *m*(*i*, *j*) genes has obtained the global ranking. Repeat the process until all genes get ranked. As shown in Fig. 3 (in Additional file 1).

## Results

Both raw count and normalized gene expression datasets are downloaded from TCGA (http://cancergenome.nih.gov/) [47], which include expression values of 20,503 genes across 102 normal samples and 779 tumor samples. Besides, gene expression datasets of Prostate adenocarcinoma containing 483 tumor samples and 51 normal samples are also downloaded from TCGA. Four thousand seven hundred twenty-six gene modules are downloaded from the website of GSEA (in Additional file 2).

Firstly, the performance of MGOGP is validated by comparing it with three module based cancer-related gene prioritization methods (MENDEAVOUR, MDK and MRWR) proposed in [34]. For comparison, the same prostate cancer network used in [34] are used, which consists of 233 genes and 1218 interactions. Modules are obtained by picking out all the GSEA modules that contain more than three genes in the prostate network after removing irrelevant module genes. Irrelevant genes are genes that are included in GSEA modules but are not included in these 233 genes. Fifteen known prostate cancer genes are obtained from OMIM (Table 1). Six genes (BRCA1, TP53, EP300, STAT3, ZFHX3, HNF1B), which are confirmed have associations with prostate cancer by Genetics Home Reference (https://ghr.nlm.nih.gov/) are used as test genes. Results are shown in Table 2.

**Table 1:** Known prostate cancer genes retrieved from the OMIM

Gene ID	Gene Symbol	Gene name
367	AR	Androgen receptor
675	BRCA2	Breast cancer type 2 susceptibility protein
3732	CD82	CD82 antigen
11200	CHEK2	Serine/threonine-protein kinase Chk2
60528	ELAC2	Zinc phosphodiesterase ELAC protein 2
2048	EPHB2	Ephrin type-B receptor 2 precursor
3092	HIP1	Huntingtin-interacting protein 1
1316	KLF6	Krueppel-like factor 6
8379	MAD1L1	Mitotic spindle assembly checkpoint proteinMAD
4481	MSR1	Macrophage scavenger receptor types I and II
4601	MXI1	MAX-interacting protein 1
7834	PCAP	Predisposing for prostate cancer
5728	PTEN	Phosphatase and tensin homolog
6041	RNASEL	2-5A-dependent ribonuclease
5513	HPC1	Hereditary prostate cancer 1

**Table 2:** Ranks of six test genes in prostate cancer gene network. They are prioritized by MDK, MRWR, Endeavour and MGOGP

Gene	MDK	MRWR	Endeavour	MGOGP
BRCA1	29	6	58	63
TP53	104	132	85	24
EP300	83	70	90	11
STAT3	39	41	88	17
ZFHX3	174	174	34	19
HNF1B	44	190	109	26
Average Rank	78	102	77	26

As shown in Table 2, all the six genes are ranked on average within top10% of all the candidate genes, which indicates the superiority of MGOGP to other three algorithms. For further comparison, we put these 21 genes together, each time we randomly select 20 different genes as known disease genes and the remaining 1 gene for test. Each run we compared the ranked positions of the 1 test gene between our method and Endeavour. Results are shown in Table 3. In Table 3 some genes do not exist, because they don’t exist in our GSEA gene modules or not exist in Endeavour database. According to Table 3, 11 of the 13 known prostate cancer-related genes and 4 of the 6 test genes have much higher ranks than these of the Endeavour. Moreover, the average ranking of these genes is 51 by MGOGP, which is better than 82 by Endeavour.

**Table 3:** Ranks of each validation gene

Gene	MGOGP	Endeavour
AR	32	30
BRCA2	29	40
CD82	169	211
CHEK2	19	35
ELAC2	64	176
EPHB2	45	165
HIP1	91	111
KLF6	88	72
MAD1L1	78	194
MSR1	60	190
MXI1	92	89
PCAP	Not Exist	Not Exist
PTEN	24	94
RNASEL	67	83
HPC1	Not Exist	Not Exist
BRCA1	46	16
TP53	5	5
EP300	11	12
STAT3	17	23
ZFHX3	59	68
HNF1B	26	12

**Fig. 4:**
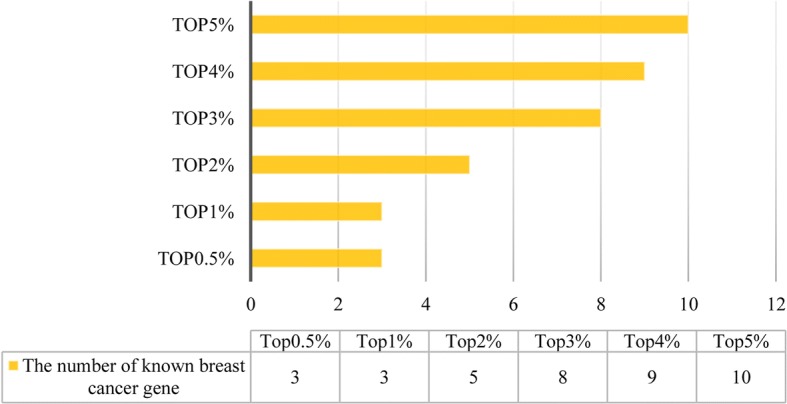
Known cancer-related gene prioritization result

Next, we use MGOGP for genome-wide breast cancer gene prioritization. We use 328 breast disease-related genes downloaded from SNP4Disease (http://snp4disease.mpibn.mpg.de/result.php) as seed genes (see Additional file 3). Ten well-known breast cancer-related genes (shown in Table 4, which are not contained in the 328 genes) are used to validate the effectiveness of our method. All GSEA gene modules are pre-processed by removing all the genes which do not have gene expression information (the final module list is supplied in Additional file 4). The result is shown in Fig. 4.

**Table 4:** Ten well-known breast cancer genes

Gene ID	Gene symbol	Gene name
672	BRCA1	Breast Cancer 1, Early Onset
675	BRCA2	Breast Cancer 2, Early Onset
7157	TP53	Tumor Protein P53
5728	PTEN	Phosphatase And Tensin Homolog
841	CASP8	Caspase 8, Apoptosis-Related Cysteine Peptidase
2263	FGFR2	Fibroblast Growth Factor Receptor 2
4214	MAP3K1	Mitogen-Activated Protein Kinase Kinase Kinase 1, E3 Ubiquitin Protein Ligas
11200	CHEK2	Checkpoint Kinase 2
472	ATM	ATM Serine/Threonine Kinase
83990	BRIP1	BRCA1 Interacting Protein C-Terminal Helicase 1

As shown in Fig. 4, all the 10 breast cancer-related genes are ranked within the top5% of the gene prioritization results. During the process, we set *S* = 1000, *ω* = 0.9 and *δ* = 0.9 (which means of the 1000 sampling results, over 90% fulfill the filter criteria). We set *υ* = 0.05 and *μ* = 0.01 as most others do [39, 41]. The performance of MGOGP under different parameter settings are supplied in Additional file 5. The top 10 ranked modules in this case study are shown in Table 5.

**Table 5:** Top 10 ranked modules

Rank	Module name	Gene number	Importance value
1	zerbini_response_to_sulindac_dn	6	0.542
2	reichert_g1s_regulators_as_pi3k_targets	8	0.523
3	sa_g2_and_m_phases	8	0.492
4	reactome_vegf_ligand_receptor_interactions	10	0.478
5	biocarta_srcrptp_pathway	11	0.461
6	honrado_breast_cancer_brca1_vs_brca2	18	0.447
7	tcga_glioblastoma_mutated	8	0.445
8	pid_vegf_vegfr_pathway	10	0.444
9	liang_silenced_by_methylation_dn	11	0.411
10	agarwal_akt_pathway_targets	10	0.410

As can be seen from Table 5, many top-ranked modules are included in well-known breast cancer pathways, such as PI3K/AKT [48] pathway and VEGF ligand-receptor pathway. The VEGF family of ligands and receptors are intimately involved in tumor angiogenesis, lymphangiogenesis, and metastasis [49]. More importantly, of the 100 genes in the top 10 ranked modules, 20 of them are contained in the KEGG breast cancer pathway (hsa05224), which is an indication of the good performance of MGOGP for cancer gene prioritization.

**Fig. 5:**
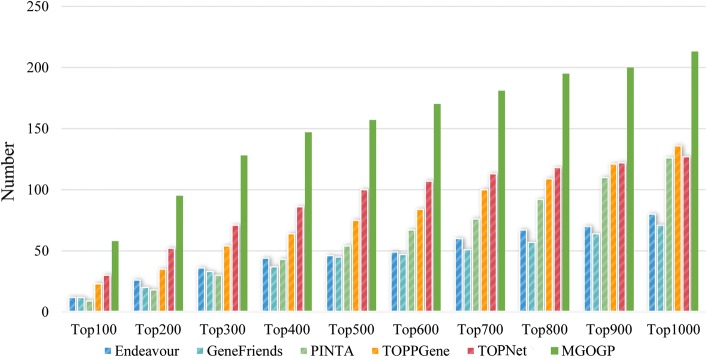
Comparison results between 6 methods. Endeavour, GeneFriends, PINTA, TOPPGene, TOppNet, and MGOGP

Next, we validate the performance of MGOGP by comparing the gene prioritization results with results obtained by methods: Endeavour [8], GeneFriends [50], PINTA [10], TOPPGene [6] and TOPNet [13]. All the methods use the same datasets and under their default parameter settings. The results are shown in Fig. 5. Brief descriptions of these methods are provided in Additional file 6. Core sourcecode of MGOGP is provided in Additional file 7. Other source codes are available from the corresponding author on reasonable request.

In Fig. 5, we count the number of breast cancer-related genes in the gene prioritization results. As is shown in Fig. 5, MGOGP outperforms other methods in detecting cancer-related genes. We use all the 328 breast disease related genes as known disease gene (Endeavour and GeneFriends used the same gene sets) and count the number of known disease genes appear in top 100–1000 prioritization results.

**Fig. 6:**
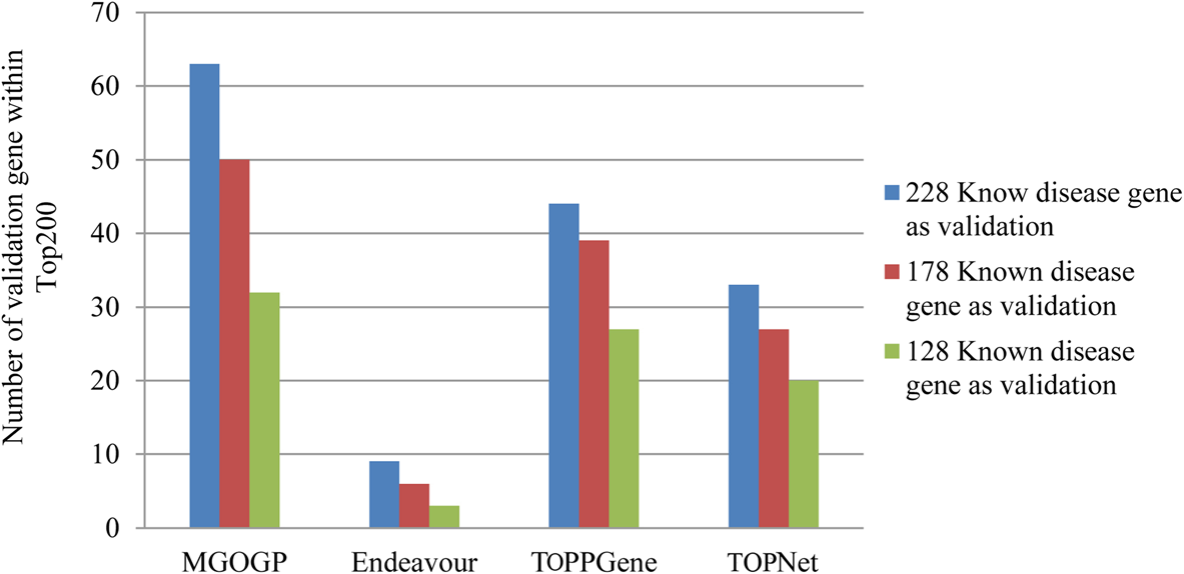
Comparison results between MGOGP, Endeavour, TOPPGene, and TOPNet with different number of known disease genes as input

To do comparison more rigorously, we further compare MGOGP to Endeavour, TOPNet and TOPPGene. Each time we randomly select 100, 150 and 200 different known disease genes from the 328 breast disease-related genes for known disease genes and others are left for test (each kind of selection repeat 100 times). We count the average number of test genes appear in Top 200 gene prioritization results. Results are shown in Fig. 6.

Finally, to further validate our method, we get the top 10 ranked genes of each method in Fig. 5. The results are shown in Table 6.

**Table 6:** Top 10 ranked genes of each method

	MGOGP	Endeavor	GeneFriends	PINTA	ToppGene	ToppNet
Top 10 gene	CCNB1IP1 CCNE2 NEK1 NRP1 CDC25C VIM PTEN VEGFB MCM2 PTGS2	SNRPF BUB3 MSH2 SSBP1 RFC4 EZH2 CENPF BLMH KIF20B BAZ1A	LURAP1L PVRL2 CYFIP1 FAM120A IL13RA1 MYO1B BCL9L NQO1 RIN2 SDC4	MGP EEF1A1 TPT1 RPS6 RPL3 RPS27 ACTB SCGB2A2 RPL11 PIP	RAD51 APEX1 SIRT2 NOC2L NEDD1 TERT EPN3 PPARGC1A NBN ATR	APP ELAVL1 NTRK1 RPA1 XPO1 EED CUL3 BARD1 HSP90AA1 NXF1
Known disease genes fall in the top 10 gene	PTEN VEGFB MCM2	MSH2 EZH2	NQO1	SCGB2A2 PIP	RAD5 TERT NBN ATR	BARD1

**Fig. 7:**
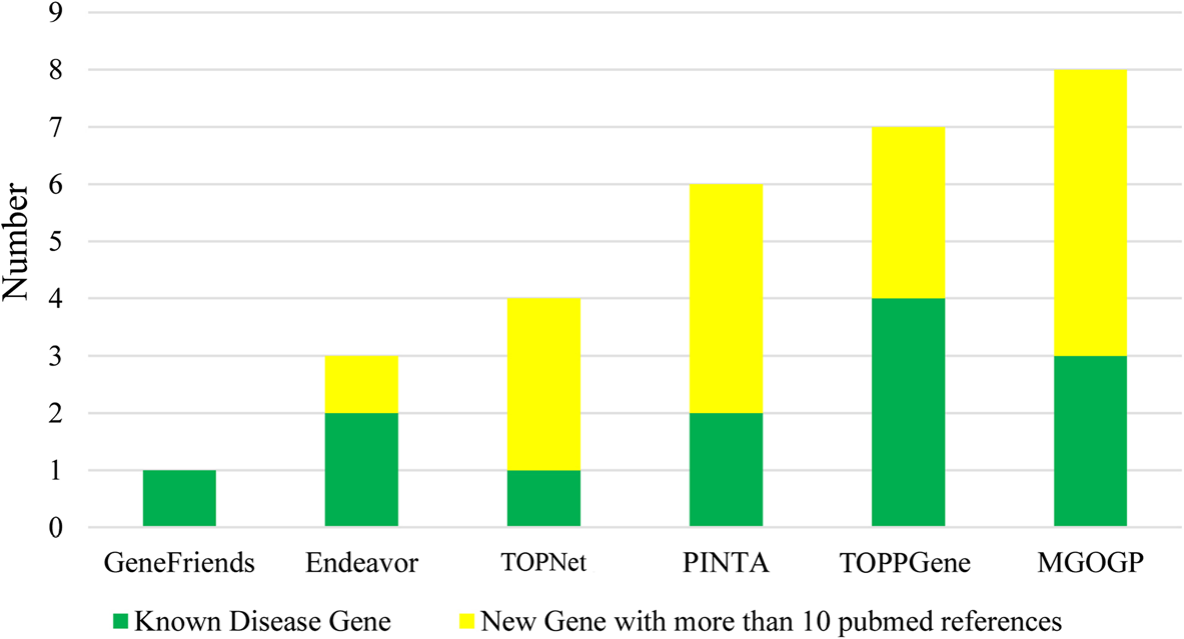
Detail statistic results of results in Table 6

In Table 6, each method is run with default parameter settings and use same training genes. Top 10 gene means the top 10 genes prioritized by each method and Known disease genes fall in the top 10 gene means genes supplied for training each method falls in the top 10 genes. Detail statistic results are shown in Fig. 7

In Fig. 7, the number of Known Disease Gene is the number of genes supplied for training each method that fall within the top 10. For example, in Table 6, PTEN, VEGFB, and MCM2 are three genes fall within the top 10 of the gene ranking result, so the number of Known Disease Gene of MGOGP in Fig. 7 is 3. For each gene within the top 10 gene ranking results of each method, we search the number of articles in PubMed mention the association between the gene and breast cancer. We count the number of genes has more than 10 PubMed article reference. As shown in Fig. 7, genes detected by MGOGP have more article supports than other methods.

## Discussion and conclusion

Results of omics experiments commonly consist of a large set of genes, while researchers usually only care about the behaviour of several genes. In this paper, a heuristic algorithm is proposed for prioritizing disease-associated genes by utilizing gene ontology annotation information and known disease-related genes as heuristic information. Different from existing methods, we propose to rank genes considering the importance of both individual genes and their affiliated modules, and utilize Gene Ontology (GO) based fuzzy measure value as well as known disease genes as heuristics, and use rank fusion strategy to obtain the global gene prioritization. Results show that MGOGP outperforms many other methods in cancer-related gene prioritization.

Different from other module-based gene prioritization methods, where modules are detected by partitioning the network using the network clustering methods, we obtain modules through gene function annotation, that is, functionally related genes are grouped into the same modules. Because gene interaction networks often suffer from the problems of high rates of false positive/negative interactions, and modules detected by network clustering algorithms often have limited accuracy, so our method is more advanced. One important difference between modules used in this study and modules detected through network partition is that no edges in our module. Instead, we use statistical methods detecting differential correlations between genes within a module, which could help avoid the preference of genes or modules that are well-researched (because currently obtained network is far from complete, the number of interactions among well-researched genes may be much more than that of newly discovered genes).

Different from module-based methods in [34], MGOGP ranks modules considering three aspects of information: module-specific gene importance, differential correlations, and importance of the module itself. In [34], the author considers the importance of a module by considering only the number of disease genes and the size of the module, which may bias toward big modules. Furthermore, gene as the major component of the module whose importance is not considered when measuring the importance of a module in [34]. While in our method, when measuring the importance of a module, we consider: the importance of the module itself, the importance of module contained genes as well as differential correlations within the module, which are the main improvements of our method.

Compared with other non-module-based prioritization methods, our algorithm also has obvious advantages. First, it is easier to find the potential pathogenic genes that cause the disease from the point of view of gene modules. Second, it takes cross-validation strategy which could guarantee the stability of the recognition results. And our method works with heuristic information which could effectively avoid the blindness of the search.

By applying MGOGP on different datasets, we demonstrate that MGOGP performs better than previous gene or network-centric methods in terms of potential disease-related genes prediction. Firstly, the performance of MGOGP is validated by comparing it with three module based cancer-related gene prioritization methods. Results show that all test genes are ranked on average within top10% of all the candidate genes. According to our results, many top-ranked modules are included in well-known cancer pathways, and top-ranked genes have more supporting PubMed articles. All of the results show that our methods perform better than the state of the art methods.

Prioritization methods are useful for assisting scientists at early research stages, and to formulate novel hypotheses of interest. In the future, one of our main goals is to see how our method behaves in other prioritization problems when using different entities and sources of data sets not covered in this study. Furthermore, we plan to study in more detail the quality of the datasets and their influence on algorithm performance, and design new methods to try to improve the results. As we all know that the methods become more mature the results will become increasingly accurate and more biologically meaningful.

## Additional files

Additional file 1: A step by step example of Rank Fusion process. This file provides an example of how to get the final gene rank. (DOCX 275 kb)

Additional file 2: GSEA gene module. This file is all the gene modules downloaded from GSEA website. (TXT 2837 kb)

Additional file 3: Breast-Cancer-Gene. This is the known breast cancer-related genes downloaded from SNP4Disease. (TXT 2 kb)

Additional file 4: Final module list. This is the refined module list after removing irrelevant genes. (TXT 2736 kb)

Additional file 5: Parameters discussion. This file discusses the performance of MGOGP under different parameter settings. (DOCX 65 kb)

Additional file 6: Brief description of gene prioritization methods. This file provides the short description of comparison methods, including their input datasets, limitations, and type. (DOCX 17 kb)

Additional file 7: Sourcecode. Some core code of our method. (TXT 5 kb)
