# Supplementary material for: MGOGP: a gene module-based heuristic algorithm for cancer-related gene prioritization
Source: BMC Bioinformatics. 2018 Jun 5;19:215. doi: 10.1186/s12859-018-2216-0 (PMC5989416; doi:10.1186/s12859-018-2216-0)
Supplement: Supplementary file 1 — A step by step example of Rank Fusion process. This file provides an example of how to get the final gene rank. (DOCX 275 kb) [file 12859_2018_2216_MOESM1_ESM.docx]

A step by step example of Rank Fusion process.

The Rank Fusion process is the process of obtaining global gene prioritization after the importance of the module and the importance of the gene within the module is measured and ranked. Assuming that we have three modules, m1, m2 and m3, their respective importance values are known, ie 0.5, 0.4 and 0.3, and the importance of the genes in the three modules and the modules has been sorted in descending order. As shown in Fig. 1 below:


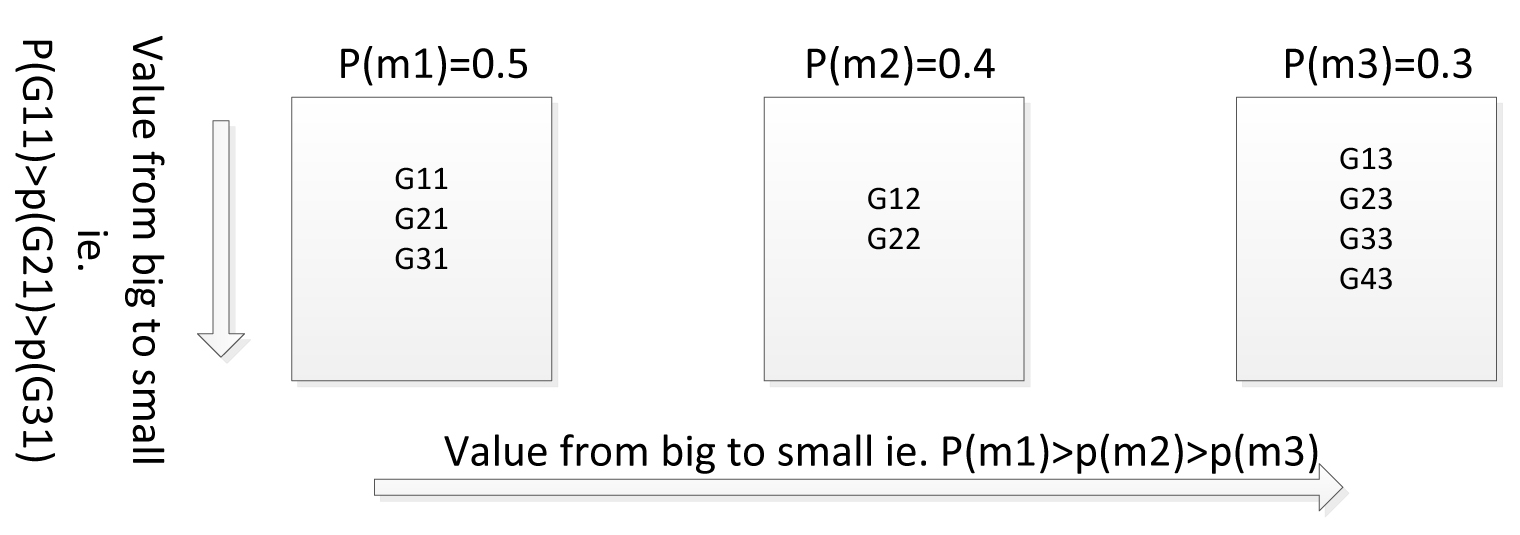


Fig. 1 Rank Fusion process example

When the algorithm is initialized, the most probable pathogenic gene G11 in the most relevant module of the disease is used as the first global pathogenic gene, then the second global gene needs to be sought. At this time:

t(2,1)=2*0.5=1 // The expected number of genes from m1 in the first two genes.

t(2,2)=2*0.4=0.8

t(2,3)=2*0.3=0.6

e(1,1)=t(2,1)-m(1,1)=1-1=0 // The expected value of the second gene from m1 is 0, m (1,1) = 1 because there is a gene G11 from module 1 in the current global ordering

e(1,2)=t(2,2)-m(1,2)=0.8-0=0.8

e(1,3)=t(2,3)-m(1,3)=0.6-0=0.6

So select G12 as the second gene.

T (3,1) = 3 * 0.5 = 1.5 // the expected number of genes from m1 in the first three genes

T (3,2) = 3 * 0.4 = 1.2

T (3,3) = 3 * 0.3 = 0.9

E (2,1) = t (3,1) -m (2,1) = 1.5-1 = 0.5 // The expected value of the third gene from m1 is 0.5, m (2,1) = 1, Because there is a gene G11 from module 1 in the current global ordering

E (2,2) = t (3,2) -m (2,2) = 1.2-1 = 0.2 // The expected value of the third gene from m 2 is 0.2, m (2,2) = 1, Because there is a gene G12 from module 1 in the current global ordering

E (2,3) = t (3,3) -m (2,3) = 0.9-0 = 0.9

So we select the gene G13 as the third gene, followed by such recursion until all the genes have been traversed. When a gene appears in multiple modules, the highest order of the gene is selected as the final sort.
