# Supplementary material for: MGOGP: a gene module-based heuristic algorithm for cancer-related gene prioritization
Source: BMC Bioinformatics. 2018 Jun 5;19:215. doi: 10.1186/s12859-018-2216-0 (PMC5989416; doi:10.1186/s12859-018-2216-0)
Supplement: Supplementary file 5 — Parameters discussion. This file discusses the performance of MGOGP under different parameter settings. (DOCX 65 kb) [file 12859_2018_2216_MOESM5_ESM.docx]

The main component of our method includes three parts, module importance measure, module gene importance measure and final rank fusion process. In module importance measure, parameters $\mu,\omega,\upsilon,\delta$are used. $\mu$is used to select differential express genes (DEGs) from DESeq2 results, which is set to 0.01 in our method and as most researchers do. We vary $\mu$from 0.001 to 0.05, the change of the percent of DEGs is shown in the following Figure 1.

Figure 1. The percent of genes (total 20503 genes in TCGA RNAseq datasets) are selected as DEGs with different $\mu$values.

After this step, we do a more rigorously DEG selection, that is only DEGs fulfill $\omega$ values are kept. We set $\omega=0.9$in MGOGP, which means after $S=1000$ runs a gene need to be detected as DEG in more than 900 times runs. We vary $\omega$ from 0.1 to 1 ($\mu=0.01$, which contains 7634 DEGs of DESeq2 result), the percent number of DEGs left are shown in the following Figure 2.

Figure 2. The percent number of the left DEGs with different $\omega$

The breast cancer RNAseq dataset we used has 102 normal samples and 779 tumor samples, each run we randomly select different tumor samples, as a result, some genes are detected as DEGs in some experiments but not in others. To select those DEGs commonly appeared in more datasets, we set$\omega=0.9$.

Because $\mu$is commonly used and well discussed in other papers, here we discussed the performance of MGOGP under different $\omega$ value. To do this, we count the number of 10 well-known breast cancer genes list in Table 4 (which are exclude from the 328 breast disease related genes as input) rank with Top 100 prioritization result gene list. As shown in Figure 3.

Figure 3. the number of 10 well known breast cancer genes within Top 100.

The reason when $\omega=1$the number of detected genes are fewer than $\omega=0.9$ maybe too less DEGs are used as input.

We use the parameter$\upsilon$ to select differential correlation between genes. The differential correlation value is calculated with fdrtool R package. We set $\upsilon=0.05$as most researchers do. In order to see the influence of $\upsilon$on the number of differential correlations within a module, we randomly select a gene module (contains 265 gene, 265*264*0.5=34980 correlations) and count the percent of differential correlations between all genes in this module under different $\upsilon$ values. The result is shown in the following Figure 4.

Figure 4. The percent of differential correlations within the selected module under different $\upsilon$

As in DEGs selection, the parameter $\delta$ is used to select differential correlations appear most frequently among each sampling result. Obviously, the higher the $\delta$ value, the more likely the differential correlations are real exist ones. In fact, the percent of differential correlation after $\delta$threshold filter follows the same distribution as in Figure 2.

As discussed above, because $\upsilon$ is commonly used and well discussed in other papers, here we only discussed the performance of MGOGP under different $\delta$ value. To do this, we use the 15 known prostate cancer genes in Table 1 as input known disease genes and we calculated the average rank of the 6 genes in Table 2. Results are shown in Figure 5.

Figure 5. The average rank of the 6 genes in Table 2 under different $\delta$value

In the module-specific gene importance measure part and global gene rank fusion part all the values are directly calculated and use, so no parameters involved in these two parts.

We vary $\mu$from 0.001 to 0.1 to see the influence of the number of differentially expressed genes on the final gene rank result, and we very$\upsilon$from 0.01 to 0.1 to see the influence of the number of differential correlations on the final gene rank result. According to our previously discussion, setting$\mu=0.01$and $\upsilon=0.05$could get proper percentage of differentially expressed genes and differential correlations. Firstly, we set $\upsilon=0.05$and we vary $\mu$from 0.001 to 0.1, to see the performance of MGOPG. Secondly, we set $\mu=0.01$and we vary $\upsilon$from 0.001 to 0.1, to see the performance of our method. We use genes in Table 4 as known breast cancer genes and we count the number of test genes (328-10=318 genes as test genes, shown in supplement file S3.) in the top 500 ranked genes under different parameter settings. Results are shown in the following Figure 6.

Figure 6. The number of test genes in the top 500 ranked genes under different parameter settings.

When we set *u=*0.001, only 48% percent of genes are select for analysis, as a result some test genes will be omitted. However, we measure gene importance to cancer not only consider gene’s differential expression but also gene’s differential correlation with other genes. In general, most cancer-related genes are differentially expressed, but some genes with small changes in expression can also be cancer-related genes. So we set $\mu=0.01$to select as many genes as possible. And we set $\upsilon=0.05$.
