# Supplementary material for: MGOGP: a gene module-based heuristic algorithm for cancer-related gene prioritization
Source: BMC Bioinformatics. 2018 Jun 5;19:215. doi: 10.1186/s12859-018-2216-0 (PMC5989416; doi:10.1186/s12859-018-2216-0)
Supplement: Supplementary file 6 — Brief description of gene prioritization methods. This file provides the short description of comparison methods, including their input datasets, limitations, and type. (DOCX 17 kb) [file 12859_2018_2216_MOESM6_ESM.docx]

Table 1 Brief Description of Gene Prioritization Methods

| Method | Brief Description | Data Sources | Limitations | type |
| --- | --- | --- | --- | --- |
| Endeavour | Endeavour is based on how similar a candidate gene is to a profile derived from genes already known to be involved in the process of interest. | coding sequence, gene expression,  Known disease genes, functional annotation, literature,  regulatory information | The quality of the data derived from multiple sources greatly affects the performance of the prioritization methods. | Gene-centric |
| PINTA | PINTA prioritizes genes based on the differential expression of their neighborhood in a genome-wide protein–protein interaction network. | gene expression and PPI networks | it does not consider the effect of gene differential expression on its functional modules. In addition, due to the defects of the PPI network, many useful genes are difficult to be excavated. | Network-centric |
| ToppGene | Extending on an earlier hypothesis that the majority of genes that impact or cause disease share membership in any of several functional relationships. ToppGene for the first time, utilizes of mouse phenotype data in human disease gene prioritization. | Gene Ontology (GO)，  Mammalian Phenotype (MP)  Pathway，  protein Domains，  PubMed，  Protein interactions，  Known disease genes,  Gene expression. | The coverage of the gene functional annotations is a limiting factor. | Datasets fusion. |
| ToppNet | ToppNet is a candidate gene prioritization method that is entirely based on protein-protein interaction network (PPIN). | human protein-protein interactions,  Known disease genes | PPI network suffers from several drawbacks as described above. | network-centric |
| GeneFriends | GeneFriends employs a RNAseq based gene co-expression network for candidate gene prioritization, based on a seed list of genes, and for functional annotation of unknown genes in human and mouse. | RNA-seq data | Selecting threshold value is   difficult in constructing gene co-expression network, besides, co-expressed genes cannot guarantee their share similar functions. | Gene-centric |
| MGOGP | MGOGP ranks genes considering both their own and their belonged module importance, and utilizes gene ontology (GO) based fuzzy measure value and known disease genes as heuristic information. | Gene expression, Known Disease Genes,  GO, GSEA gene module. | The coverage of the gene functional annotations is a limiting factor. | Datasets fusion. |
